# FROM SALTWATER TO LAND: BIRD ASSEMBLAGES AND NEW RECORDS IN THE SOUTHERN ANSENUZA WETLAND

**DOI:** 10.21203/rs.3.rs-8523975/v1

**Published:** 2026-02-18

**Authors:** Gabriel Barco, Ezequiel Vivas, Adrian Díaz, David L. Vergara-Tabares

**Affiliations:** 1. Laboratorio de Arbovirus. Instituto de Virología “J. M. Vanella” (FCM) - UNC/CONICET, Enfermera Gordillo Gómez, X5000, Córdoba, Argentina.; 2. Proyecto Ansenuza, Aves Argentinas. Asociación Ornitológica del Plata, Matheu 1246/8, C1249, Buenos Aires, Argentina.; 3. Instituto de Diversidad y Ecología Animal (FCEFyN) - UNC/CONICET, Av. Vélez Sarsfield 299, X5000, Córdoba, Argentina.

**Keywords:** wetlands, habitats, update, vagrants

## Abstract

Wetlands are ecosystems of critical importance for human health and well-being. Despite this, they remain undervalued and face significant conservation challenges. Birds are a distinctive taxonomic group in these environments contributing substantially to ecosystem function. In the continental interior of South America, the Dulce River marshes and Mar de Ansenuza Lake form an extensive wetland of international importance, particularly for migratory birds. We propose to analyze bird communities composition in relation to environmental heterogeneity in the southern sector of the Ansenuza lake and assess whether the wetland functions as a relevant point for vagrant birds. We used presence–absence data of birds across various environments, combining field observations with historical citizen science records, to conduct dissimilarity analyses and hierarchical clustering based on average linkage. Additionally, we identified and evaluated novel species recorded over the past 10 years. Bird communities showed significant differences among environments (R = 0.66, p = 0.001). Ordination analysis strongly indicated two groups, the terrestrial environment hosting the highest species richness (53%), and the aquatic group showing the greatest species exclusivity. Of a total of 345 species recorded, 24 were newly displayed diverse migratory behaviors. Our results highlight the increasing use of the wetland by birds and suggest that it may function as an important refuge and critical resource hub for wide-ranging avian species. These findings underscore the need for both habitat-specific and integrated conservation strategies.

## 1. Introduction

Wetlands are ecosystems found worldwide, characterized by the presence of water, either temporarily or permanently (Ramsar 2018). These ecosystems play a crucial role in human health and well-being (Maltby and Acreman 2011). They provide a wide range of benefits, including water retention and release for consumption and irrigation, carbon sequestration and storage, food security, medicinal resources, and aesthetic, cultural, and social values (Wenny et al. 2011; Xu et al. 2020). Despite these significant contributions, most studies agree that wetlands are undervalued and face serious conservation challenges. In particular, salt lakes are among the most threatened wetlands globally (Moomaw et al. 2018; Davidson et al. 2019). These ecosystems are increasingly affected by climate change, pollution, watershed canalization, water depletion, and the expansion of agricultural and urban areas (Jellison et al. 2008; Saccò et al. 2021).

Birds are a distinctive taxonomic group within the rich biodiversity that wetlands support, characterized by their richness and abundance. Approximately 10% of global bird species depend on these environments at some stage of their life cycle (Williamson et al. 2013; Rannestad et al. 2015). Birds also contribute significantly to ecosystem services, benefiting both wetlands and human communities. Their roles include nutrient cycling, stimulating primary production, pest control, plant dispersal, promoting ecotourism, and providing cultural and artistic value, among others (Green and Elmberg 2014). In addition, birds often may respond to environmental changes and presence of disturbance sources (Frota et al. 2022; Taft et al. 2002). Assessing bird richness and diversity in these ecosystems is essential for evaluating their health and conservation status (Dunn et al. 2006).

**Fig. 1:**
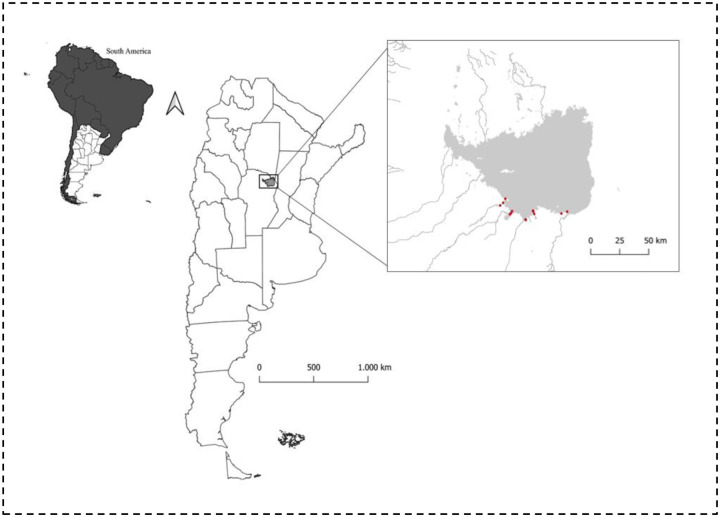
Geographic location of the Ansenuza lake. Shows (red dots) study areas in the coast south of Mar de Ansenuza lake

In South America, wetlands listed as Wetlands of International Importance cover 3.2% of the continent’s surface area (Ramsar 2023). In the interior of the continent, at temperate latitudes, Dulce River marshes and Mar de Ansenuza Lake (MLA) form a vast wetland ecosystem spanning 9,960 km^2^. This site serves as a crucial convergence point for birds from various migratory routes across the Americas (Acosta et al. 2006). The lake is primarily fed with fresh water from the north by the Dulce River and its marshes and from the south by the Suquía and Xanaes Rivers (Fig. 1). It is known that at the mouths of these rivers, the transition from fresh water to salt water creates estuaries that promote the highest concentration of biodiversity in the entire wetland (Bucher 2019; Nores 2024).

Due to the annual gathering of more than 500,000 shorebirds, this wetland is recognized as a key site for the conservation of avian diversity, particularly migratory species. It has been designated a Hemispheric Bird Site by the Western Hemisphere Shorebird Reserve Network, an Important Bird Area by BirdLife International (Torres and Michelutti 2007), and part of the vast territory was declared National Park in 2022. Historical records of documented population counts of 42,800 juvenile and 100,000 adult *Phoenicopterus chilensis*, 250,000 *Phalaropus tricolor*, and over 300,000 *Egretta thula* individuals (Torres and Michelutti 2006). The region also is one of the most important wintering areas for *Phoenicoparrus jamesi* and *Phoenicoparrus andinus* during their winter migration (Derlindati et al. 2024). These facts highlight the importance of the site for waterbirds conservation but the biodiversity of the surrounding environments remains understudied.

In recent decades, most avian studies at this site have focused exclusively on aquatic communities, leaving significant knowledge gaps regarding bird assemblages in other environments. Descriptions of bird assemblages along different environments associated with Ansenuza wetland are basic information needed to guide and contribute for conservation planning. Here, we propose to analyze bird communities composition and how these assemblages are associated with respect to the environmental heterogeneity. Using our own field data together with records from the eBird citizen science platform, we aim to describe bird assemblages across different environments, assess their similarities, and identify records of vagrant species.

## 2. Materials and methods

### 2.1. Study area

The study area is located in the southern sector of Ansenuza Lake, in northeastern Córdoba Province, Argentina (30°54’S, 62°45’W). The wetland landscape is composed of various environmental units, including patches of agricultural and livestock fields, forests, halophytic shrublands, steppes, flooded savannas, and aquatic environments (Menghi 2006). From a physiognomic-floristic perspective, the dominant vegetation in this region consists of *Prosopis nigra* and *Prosopis alba* forests, with *Aspidosperma quebracho-blanco*, forming part of the “Sclerophyllous Forest with *Prosopis nigra* and *Prosopis alba”* vegetation unit within the Phytogeographic Province of Espinal (Oyarzabal et al. 2018). Currently, the area is almost entirely deforested, with only small fragments of the original forest remaining. These forest remnants are associated with elevated soils at the extreme end of a soil salinity gradient, transitioning into marshy ecotones dominated by halophytic shrublands before reaching the lake’s coastline (Bucher et al. 2006). The terrain elevation ranges from 60 to 80 m above sea level. Annual precipitation varies between 800 and 900 mm, primarily concentrated in the summer months. The mean annual temperature is 19°C, with an average maximum of 32°C in January and a minimum of 3.5°C in July (Bucher 2019).

### 2.2. Bird surveys and data compilation

**Fig. 2:**
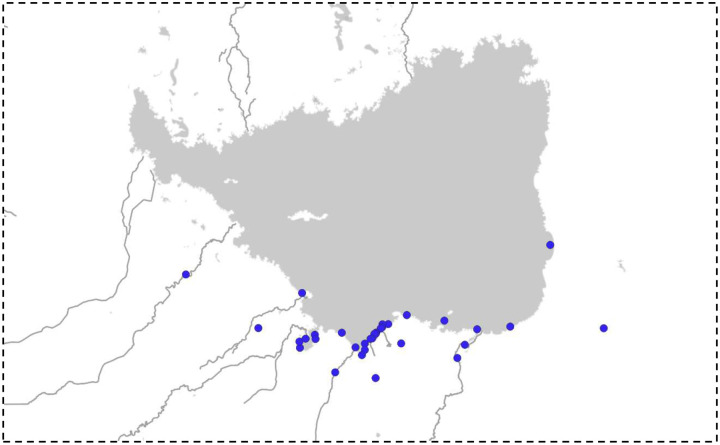
Sites of interest with abundant records of citizen science birds used to form a single updated list. Data obtained from eBird.

We selected five gradient areas, each separated by a minimum of 8 km. Within each area, we established point counts across three distinct environments: forest, halophytic shrubland, and coastal zones. In forest and halophytic shrublands, we established 16 fixed-radius observation points (30 m) systematically distributed at least 200 m apart, totaling 80 point counts per environment. In aquatic environments, we conducted four observation points, each covering a 300-meter stretch of coastline, resulting in a total surveyed length of 3.6 km. During the early morning hours, we recorded all birds detected by sight and sound over a 10-minute period at each point (Ralph et al. 1998). Surveys were conducted in 2023 during autumn and summer. Additionally, to compile a comprehensive bird species list for the southern sector of the region, we integrated our field data with records from the citizen science platform eBird, encompassing 33 sites of interest within the same region (Fig. 2).

We also analyzed the presence and absence of birds across the same environmental categories described previously, with the addition of two categories: agroecosystems and estuaries. The bird list included order, family, scientific and common name, migratory behavior and the type of environment in which each species was observed (Supplementary material 1). The scientific nomenclature and taxonomic classification of species followed the 2023 version of the eBird/Clements Checklist (Clements et al. 2023), as adopted by the eBird platform. Additionally, we identified as novel records those species observed for the first time in the southern region of the lake within the last decade, between 2015 and 2025. For each species, we report the known migratory patterns based on Salvador et al. (2016), Capllonch (2018), Jahn et al. (2020) and online data from eBird. We also assessed whether these records represented novel occurrences for Argentina, Córdoba province or the southern region of the lake. We also mentioned in which season these species were recorded and finally, we considered each species’ conservation status according to the IUCN Red List.

### 2.3. Data Analyses

Using field-collected data, we constructed a presence-absence matrix of bird species across different environments. Similarity analysis (ANOSIM) was performed using the *vegan* package, employing Jaccard’s dissimilarity index to assess community differences. Additionally, we examined similarities among environments and bird assemblage composition using hierarchical clustering (UPGMA) based on Jaccard’s qualitative similarity index. To assess the robustness of the resulting clusters, a bootstrap analysis with 10000 replicates was conducted. All analyses were conducted in R v.4.3.1 (R Core Team 2016). To answer the question of whether the lagoon functions as a target point for vagrant birds, we analyzed historical bird lists for the region and identified those species that have been incorporated in the last 10 years between 2015–2025.

## 3. Results

### 3. 1. Full list and bird assemblages among environments

**Fig. 3:**
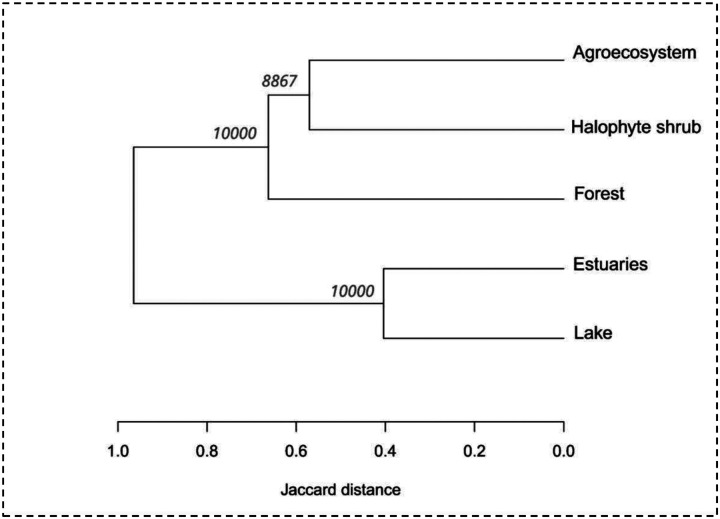
Dendrogram resulting from classification analysis of bird assemblages across different habitats. Bootstrap support values are indicated at the nodes.

**Fig. 4:**
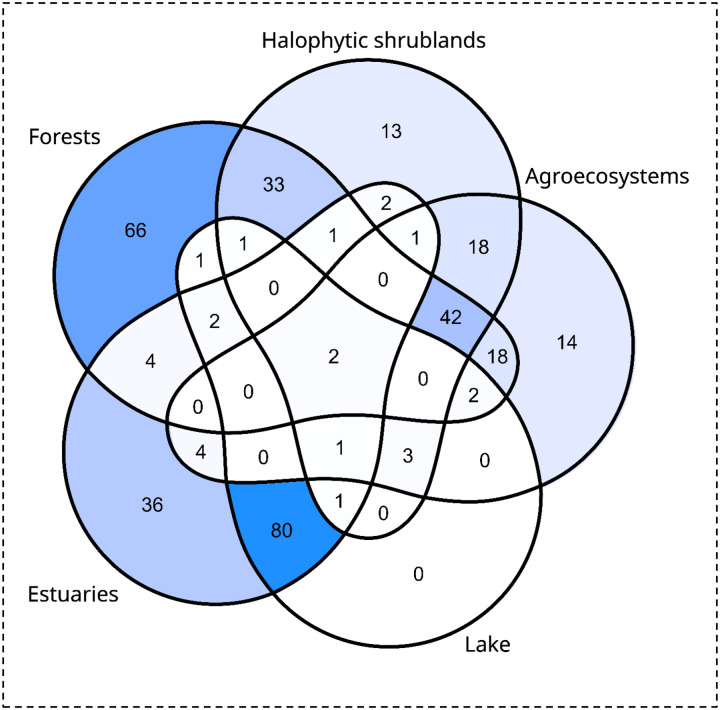
Number of species recorded in each environment, distinguishing between shared and exclusive species. The intensity of the blue color is proportional to the number of species.

Based on our field observations in different environmental categories, the non-parametric analysis revealed significant differences in bird communities across environments (ANOSIM, R = 0.66, *p* = 0.001). The unified list of our data with citizen science data for the region comprised 345 species, distributed across 25 orders and 58 families. Among Passeriformes, the most represented families were Tyrannidae (47 species), Thraupidae (29 species), and Furnariidae (26 species). For non-Passeriformes, the most diverse families were Scolopacidae (20 species), Anatidae (22 species), and Laridae (16 species). Approximately 60% of the present species have migratory behavior (see Material Suplementary 1). Graphical exploration of the ordination analysis revealed how the environments were arranged according to the dissimilarity of their bird assemblages (Fig. 3). The bootstrap analyses showed maximum support (100%) for two groups, terrestrial (forests-halophytic shrublands-agroecosystem) and aquatic groups (lakes-estuaries). In the first group, the three environments share 42 species (representing ~12% of total recorded species) and in the second group, the two environments share 80 species (~23% of total species; Fig. 4). All environmental categories showed a high degree of species shared with other environments and even presented exclusive species, with the exception of lakes, that all his species were also observed in estuaries (Fig. 4). The forests showed the greatest richness of birds (50%) and the highest number of exclusive species (38%), followed by estuaries (39% and 27% respectively). Only two species were recorded in all environments, *Caracara plancus* and *Daptrius chimango*.

### 3.2. Record of new species

We identified species records from the past decade that had not been previously documented in the region, resulting in a total of 24 newly recorded species (Table 1). These newly identified species exhibited diverse migratory behaviors, including resident species (e.g., *Donacobius atricapilla*, remaining year-round in the same area), temperate–tropical migrants (e.g., *Elaenia spectabilis*, migrating seasonally between temperate and tropical regions), temperate–austral migrants (e.g., *Zonibix modestus*, moving between temperate and southern latitudes), temperate–altitudinal migrants (e.g., *Streptoprogne zonaris*, shifting between elevations according to season), and temperate–longitudinal migrants (e.g., *Oreophollus ruficollis*, migrating across longitudinal gradients) and also species with a Palearctic-African migratory history (e.g., *Chlidonias leucopterus*).

## 4. Discussion

The contribution presented in this study strengthened the bird’s inventories in the region. It is also clear that the data continuously generated by citizen science and collected through web platforms are valuable for knowledge of the nature and dynamic of species (Pocock et al. 2019; Santos et al. 2023). In our case, because species detectability may vary, increased ornithological explorations at a given site helped to detect certain rare or very cryptic species that may be underestimated. Many sites, mainly terrestrial, have the advantage of being easily accessible to any birdwatchers, resulting in a large volume of data and increased reliability. The richness of birds for this southern region of wetland alone concentrated 75% of the species mentioned for Córdoba province (Salvador et al. 2016). This gives reference to the important contribution that this region makes to the diversity of avifauna. Over half of species have migratory behavior, reaffirming this wetland as a key connectivity node for migratory routes and as a fundamental habitat in the territorial dynamics of species (Bucher et al. 2006; Capllonch 2018).

Ordination analysis showed there is more than one assemblage of bird communities present in the region. At first glance, two main clusters or groups have been distinguished, terrestrial and aquatic groups. The bootstrap support values within the terrestrial group demonstrate the strong possibility of dissimilarity between forests and halophyte agroecosystems/shrublands. Studies focused solely on these terrestrial environments would be necessary to corroborate this dissimilarity. The results obtained are consistent with other studies showing that, at local scales, vegetation structure and composition are strong factors shaping bird community assemblages (De Stefano et al. 2012; Özkan et al. 2013). Particularly in landscapes with strong anthropic pressure, it should not be lost sight of the fact that changes in vegetation structure can lead to changes and impoverishment in the composition of bird communities (Skowno and Bond 2003). In the face of the question of wetland bird conservation, we believe that, given the important contribution of each environment to the richness of wetland birds, it is advisable to plan specific strategies for each environment and integrated strategies.

The identification of species previously unrecorded in the area over the past decade raises new questions about the possible drivers behind these new observations. Nores (2011, 2024) published how the variation in water level and salinity of the lake influenced the detection and abundance of some waterfowl and shorebirds. It also highlights records of Atlantic coastal species in Mar de Ansenuza as being outside their usual migratory pathways (Nores 2024). In this research we detected that the new records exhibit diverse life history traits and migratory behaviors makes it difficult to clarify a clear pattern. It is well known that shifting species distributions can be driven by multiple factors, including climatic, biologic, geography and stochastic processes (Pigot et al. 2010). In the case of migratory birds the success of migration often depends on climatic conditions and anthropic influences. For example, light pollution can alter migratory routes or force nocturnal migrants to use different stopover sites. Likewise, the nutritional status of migrants can determine whether they change stopover sites or adjust the duration of stopovers (Anderson et al. 2019; Schmaljohann et al. 2022). Therefore, the wetland ecosystem of Mar de Ansenuza, and particularly the extensive water surface of its lagoon, likely acts as a large-scale “target effect” for birds in vagrant condition. This effect may provide alternative stopover site for migratory birds such as *C. minutilla, C. leucopterus* and *O. ruficollis*; or it may also offer new areas for species expanding from contiguous distributions, such as *D. atricapilla, A. obscura, P. ruber* and *T. doliatus*. Although the exact parameters birds use when selecting new sites are not fully understood, it is clear that both resource availability and conspecific interactions can play decisive roles (Muller et al. 1997). Further species-specific studies and long-term monitoring will be needed to determine whether these records reflect true range expansion and to clarify the role of Mar de Ansenuza as a key target site for vagrant birds.

## 4. Conclusions

In the southern region of the wetland, we observed an increase in the number of bird species using the site, either seasonally or year-round. Additionally, our findings demonstrate clear differentiation among environmental types based on bird community composition. This evidence supports the need to extend conservation and management plans beyond aquatic boundaries. The newly recorded bird species over the past decade suggest that, although further empirical validation is needed, the lake and its surroundings may serve as an important refuge and resource hub for wandering birds. Similar research would need to be extended to other regions in order to achieve greater clarity on population dynamics at both large and small scales.

## Supplementary Material

Supplementary Files

This is a list of supplementary files associated with this preprint. Click to download.

- Supplementarymaterial1.pdf

## Figures and Tables

**Table 1.** New species registered south of Mar de Ansenuza Lake in the last decade (2015–2025). Season observed: **Wi**, winter; **Sp**, sprinter; **Su**, summer; **Fa**, fall. Novel record for: **Reg,** regional; **Arg**, Argentine country; **Cba**, Cordoba province. State IUCN: **VU**, vulnerable; **LC**, least concern; **NT**, near threatened.

Scientific name	Migrating behavior	Season observed	Novel record for	State IUCN	Reference
*Asemospiza obscura*	Temperate-tropical	Su	Cba	LC	1,2
*Calidris ferruginea*	Neartic	Sp	Arg	VU	3
*Calidris minutilla*	Neartic	Sp	Arg	NT	4
*Chlidonias leucopterus*	Palearctic-african	Wi- Sp	Arg	LC	5, 6,7
*Columbina talpacoti*	Temperate-tropical	Su	Reg	LC	8,9
*Donacobius atricapilla*	Resident	Sp	Reg	LC	10
*Egretta caerulea*	Temperate-tropical	Wi	Cba	LC	11,12
*Elaenia spectabilis*	Temperate-tropical	Sp-Su	Reg	LC	13,14
*Leucophaeus atricilla*	Neartic	Sp	Arg	LC	15,16
*Nomonyx dominicus*	Resident	Su	Cba	LC	17,18
*Oreophollus ruficollis*	Temperate-longitudinal	Au	Reg	LC	19
*Phacellodomus ruber*	Temperate-tropical	Sp- Su	Cba	LC	20,21,22
*Phaetusa simplex*	Temperate-tropical	Sp- Su	Reg	LC	23,24
*Phalaropus fulicarius*	Nearctic	Sp	Reg	LC	25
*Piaya cayana*	Resident	Sp	Cba	LC	26,27
*Pluvialis squatarola*	Nearctic	Sp-Su	Cba	VU	28
*Rhopospina fruticeti*	Temperate-austral	Fa	Cba	LC	29,30
*Streptoprocne zonaris*	Temperate-altitudinal	Sp	Reg	LC	31,32
*Thalasseus sandvicensis*	Temperate-tropical	Wi- Sp	Reg	LC	33,34
*Thamnophilus doliatus*	Resident	Su	Cba	LC	35,36
*Turdus chiguanco*	Temperate-altitudinal	Au-Sp-Su	Reg	LC	37
*Tyrannus tyrannus*	Nearctic	Sp-Su	Cba	LC	38
*Xema sabini*	Nearctic	Sp	Cba	LC	39,40
*Zonibyx modestus*	Temperate-austral	Fa-Wi	Cba	LC	19,41

Reference: (1) Vivas 2024; (2) Capllonch 2018; (3) Toledo et al. 2018; (4) Martínez-Curci et al. 2018; (5) Gochfel et al. 2020a; (6) Gaia 2023; (7) Meurzet 2020; (8) Vivas 2024; (9) Hart 2020; (10) Moriondo 2023; (11) Ansenuza 2016; (12) Rodgers and Smith 2020; (13) Vivas 2022; (14) Hosner and Kirwan 2020; (15) Herrera and Tejerina 2022; (16) Burger 2020; (17) Eitniear 2020; (18) Montani 2020; (19) Ansenuza 2018; (20) Remsen 2020; (21) Mendoza 2020; (22) García-Loyola 2019; (23) Ansenuza 2015; (24) Gochfel et al. 2020b; (25) Gaia 2023; (26) Ansenuza 2020; (27) Fitzgerald et al. 2020; (28) Wendeler 2022; (29) Jaramillo and Pantoja 2024; (30) Schlemmer 2018; (31) Vivas 2023; (32) Roper 2020; (33) Shealer et al. 2020; (34) Nores 2024; (35) Beltroco and Capovilla 2020; (36) Kolof and Menill 2020; (37) Caverzasi 2020; (38) Ecoregistros 2025; (39) Loredo et al. 2023; (40) Day et al. 2020; (41) Arcaya-Orrego et al. 2025.

## Data Availability

The datasets generated during and/or analysed during the current study are available in the Figshare repository, https://doi.org/10.6084/m9.figshare.31017988.v1

## References

[R1] Acosta OO, Torres-Dowdall JR, Martin E, Lascano E (2006). Shorebirds. In: Bucher EH (ed) Bañados del Río Dulce and Laguna Mar Chiquita (Córdoba, Argentina). National Academy of Sciences, Córdoba, Argentina, pp 263–275.

[R2] Anderson AM, Duijns S, Smith P, Friis C, Nol E (2019). Migration Distance and Body Condition Influence Shorebird Migration Strategies and Stopover Decisions During Southbound Migration. Front. Ecol. Evol. 7:251. 10.3389/fevo.2019.00251

[R3] Ansenuza M (2015). eBird: Online database of bird distribution and abundance [web application]. Cornell Lab of Ornithology, Ithaca, New York. eBird checklist: https://ebird.org/checklist/S25957087 (accessed 26 June 2025).

[R4] Ansenuza M (2016). eBird: Online database of bird distribution and abundance [web application]. Cornell Lab of Ornithology, Ithaca, New York. eBird checklist: https://ebird.org/checklist/S30634746 (accessed 26 April 2025).

[R5] Ansenuza M (2018). eBird: Online database of bird distribution and abundance [web application]. Cornell Lab of Ornithology, Ithaca, New York. eBird checklist: https://ebird.org/checklist/S46307542 (accessed 10 July 2025).

[R6] Ansenuza M (2020). eBird: Online database of bird distribution and abundance [web application]. Cornell Lab of Ornithology, Ithaca, New York. eBird checklist: https://ebird.org/checklist/S76173175 (accessed 12 July 2025).

[R7] Arcaya-Orrego N, Pantoja-Maggi V, Pyle P, Boesman P, Wiersma P, Kirwan G (2025). Rufous-chested Dotterel (*Zonibyx modestus*), version 2.0. In Birds of the World (Smith M. G., Editor). Cornell Lab of Ornithology, Ithaca, NY, USA. 10.2173/bow.rucdot1.02

[R8] Beltrocco EL, Capovilla P (2020). Barred Antshrike (*Thamnophilus Doliatus*) in Sauce Viejo, Santa Fe. Nuestras Aves, no. 65 (December): 66. 10.56178/na.vi65.146

[R9] Bucher EH (2019). The Mar Chiquita Salt Lake (Córdoba, Argentina). Springer International Publishing, pp 1–149

[R10] Bucher EH, Gavier Pizarro G, Curto ED (2006). Geographical synthesis. In: Bucher EH (ed) Bañados del río Dulce y laguna Mar Chiquita (Córdoba, Argentina). National Academy of Sciences, Córdoba, Argentina, pp 15–27.

[R11] Burger J (2020). Laughing Gull (*Leucophaeus atricilla*), version 1.0. In Birds of the World (Rodewald P. G., Editor). Cornell Lab of Ornithology, Ithaca, NY, USA. 10.2173/bow.laugul.01

[R12] Caverzasi H (2020). eBird: Online database of bird distribution and abundance [web application]. Cornell Lab of Ornithology, Ithaca, New York. eBird checklist: https://ebird.org/checklist/S72072728 (accessed 2 June 2025).

[R13] Capllonch P (2018). An overview of bird migrations in Argentina. El hornero, 33(1), 01–18.

[R14] Clements JF, Rasmussen PC, Schulenberg TS, Iliff MJ, Fredericks TA, Gerbracht JA, Lepage D, Spencer A, Billerman SM, Sullivan BL, Wood CL (2023). The eBird/Clements checklist of Birds of the World: v2023. https://www.birds.cornell.edu/clementschecklist/introduction/updateindex/october-2025/2025-citation-checklist-downloads/

[R15] Davidson NC, Dinesen L, Fennessy S, Finlayson CM, Mcinnes R (2019). Trends in the ecological character of the world’s wetlands. Marine and Freshwater Research, 71 (1), pp 127.

[R16] Day RH, Stenhouse IJ, Gilchrist HG (2020). Sabine’s Gull (*Xema sabini*), version 1.0. In Birds of the World (Billerman S. M., Editor). Cornell Lab of Ornithology, Ithaca, NY, USA. 10.2173/bow.sabgul.01

[R17] Derlindati EJ, Arengo F, Michelutti M, Romano MC (2024). A review of the ecology and conservation of the Andean Flamingo *Phoenicoparrus andinus* and Puna Flamingo *P. jamesi* in South America. Bird Conservation International, 34, e37.

[R18] De Stefano K, Merler JA, Magnano AL, Nanni AS, Kandus P, Quintana RD (2012). Relationship between environmental heterogeneity and the distribution pattern and species richness of birds in two landscape units of the Paraná River Delta. Ornitol. Neotrop. 23, pp 169–84.

[R19] Dunn EH, Bart J, Collins BT, Craig B, Dale B (2006). Monitoring bird populations in small geographic areas. Occasional Paper of the Canadian Wildlife Service (SPEC. ISS.):1–59.

[R20] EcoRegistros (2025). Eastern Kingbird (*Tyrannus tyrannus*). Species account. https://www.ecoregistros.org (accessed 21 April 2025).

[R21] Eitniear JC (2020). Masked Duck (Nomonyx dominicus), version 1.0. In Birds of the World (Billerman S. M., Editor). Cornell Lab of Ornithology, Ithaca, NY, USA. 10.2173/bow.masduc.01

[R22] Fitzgerald J, Schulenberg T, Seeholzer G (2020). Squirrel Cuckoo (Piaya cayana), version 1.0. In Birds of the World (Schulenberg T. S., Editor). Cornell Lab of Ornithology, Ithaca, NY, USA. 10.2173/bow.squcuc1.01

[R23] Frota AVB, Vitorino BD, Almeida SM, da Silva Nunes JR, da Silva CJ (2022). Bird dependence on wetlands determines functional responses to flood pulse in the Brazilian Pantanal. Ornithology Research, 30(3), pp 190–203.

[R24] García-Loyola E (2019). eBird: Online database of bird distribution and abundance [web application]. Cornell Lab of Ornithology, Ithaca, New York. eBird checklist: https://ebird.org/checklist/S52956537 (accessed 2 July 2025).

[R25] Gochfeld M, Burger J, Christie D, Kirwan GM, Garcia E (2020)a. White-winged Tern (*Chlidonias leucopterus*), version 1.0. In Birds of the World (del Hoyo J., Elliott A., Sargatal J., Christie D. A., and de Juana E., Editors). Cornell Lab of Ornithology, Ithaca, NY, USA. 10.2173/bow.whwter.01

[R26] Gochfeld M, Burger J, Kirwan G, Garcia E (2020)b. Large-billed Tern (*Phaetusa simplex*), version 1.0. In: Birds of the World (del Hoyo J., Elliott A., Sargatal J., Christie D. A., and de Juana E., Editors). Cornell Lab of Ornithology, Ithaca, NY, USA. 10.2173/bow.labter1.01

[R27] Green AJ, Elmberg J (2014). Ecosystem services provided by waterbirds. Biological reviews, 89(1), 105–122. 23786594 10.1111/brv.12045

[R28] Hart JA (2020). Ruddy Ground Dove (*Columbina talpacoti*), version 1.0. In Birds of the World (Schulenberg T. S., Editor). Cornell Lab of Ornithology, Ithaca, NY, USA. 10.2173/bow.rugdov.01

[R29] Herrera VN, Tejerina V (2022). A New Species for the Avifauna of Argentina: the Black-headed Gull (*Leucophaeus Atricilla*) in Jujuy. Nuestras Aves, no. 67 (December). 10.56178/na.vi67.11.

[R30] Hosner P, Kirwan G (2020). Large Elaenia (*Elaenia spectabilis*), version 1.0. In Birds of the World (del Hoyo J., Elliott A., Sargatal J., Christie D. A., and de Juana E., Editors). Cornell Lab of Ornithology, Ithaca, NY, USA. 10.2173/bow.larela1.01

[R31] Jahn AE, Cueto VR, Fontana CS, Guaraldo AC, Levey DJ, Marra PP, Ryder TB (2020). Bird migration within the Neotropics. The Auk, 137(4).

[R32] Jaramillo A, Pantoja V (2024). Mourning Sierra Finch (*Rhopospina fruticeti*), version 1.2. In Birds of the World (Smith M. G., Editor). Cornell Lab of Ornithology, Ithaca, NY, USA. 10.2173/bow.mosfin1.01.2

[R33] Jellison R, Williams W, Timms B, Alcocer J, Aladin N (2008). Salt lakes: values, threats and future. In Aquatic ecosystems: trends and global prospects, pp 94–110.

[R34] Koloff J, Mennill DJ (2020). Barred Antshrike (*Thamnophilus doliatus*), version 1.0. In Birds of the World (Schulenberg T. S., Editor). Cornell Lab of Ornithology, Ithaca, NY, USA. 10.2173/bow.barant1.01

[R35] Loredo MA, Zanoletti M, Paulos A, Crinigan C, Aldunate S (2023). First Record of the Sabine’s Gull (*Xema Sabini*) in Argentina. Nuestras Aves, no. 66 (February). 10.56178/na.vi66.54

[R36] Maltby E, Acreman MC (2011). Ecosystem services of wetlands: pathfinder for a new paradigm. Hydrological Sciences Journal, 56(8), 1341–1359.

[R37] Martínez-Curci NS, Pretelli M, Cavalli M, Isacch J, Loredo M (2018). First record of Least Sandpiper *Calidris minutilla* for Buenos Aires province and review of its status in Argentina. Wader Study, 125: 1.

[R38] Mendoza J (2020). eBird: Online database of bird distribution and abundance [web application]. Cornell Lab of Ornithology, Ithaca, New York. eBird checklist: https://ebird.org/checklist/S77251103 (accessed 18 July 2025).

[R39] Menghi M (2006). Vegetation. In: Bucher EH (ed) Bañados del Río Dulce and Mar Chiquita Lake (Córdoba, Argentina). National Academy of Sciences, Córdoba, Argentina, pp 173–189.

[R40] Montani S (2020). eBird: Online database of bird distribution and abundance [web application]. Cornell Lab of Ornithology, Ithaca, New York. eBird checklist: https://ebird.org/checklist/S102481114 (accessed 21 July 2025).

[R41] Moomaw WR, Chmura G, Davies G, Finlayson C, Middleton B, Natali S, Sutton-Grier A (2018). Wetlands in a changing climate: science, policy and management. Wetlands, 38(2), 183–205.

[R42] Moriondo D (2023). eBird: Online database of bird distribution and abundance [web application]. Cornell Lab of Ornithology, Ithaca, New York. eBird checklist: https://ebird.org/checklist/S173349680 (accessed 2 April 2025).

[R43] Muller KL, Stamps JA, Krishnan VV, Willits NH (1997). The effects of conspecific attraction and habitat quality on habitat selection in territorial birds (*Troglodytes aedon*). The American Naturalist, 150(5), 650–661. 10.1086/286087 18811306

[R44] Nores M (2011). Long-term waterbird fluctuations in Mar Chiquita Lake, central Argentina. Waterbirds, 34(3), 381–388.

[R45] Nores M (2024). Waterbird fluctuations in Mar Chiquita Lake, central Argentina: the last 13 years. Waterbirds, 46(2–4), 199–204.

[R46] Oyarzabal M, Clavijo J, Oakley L, Biganzoli F, Tognetti P, Barberis I, (2018). Vegetation units of Argentina. Ecología austral, 28(01).

[R47] Özkan K, Svenning J, Jeppesen E (2013). Environmental species sorting dominates forest-bird community assembly across scales. Journal of Animal Ecology, 82(1), 266–274. 22849355 10.1111/j.1365-2656.2012.02019.x

[R48] Pigot AL, Owens I, Orme C (2010). The environmental limits to geographic range expansion in birds. Ecology Letters, 13(6), 705–715. 20412281 10.1111/j.1461-0248.2010.01462.x

[R49] Pocock MJ, Roy HE, August T (2019). Developing the global potential of citizen science: Assessing opportunities that benefit people, society and the environment in East Africa. Journal of Applied Ecology, 56(2), 274–281.

[R50] Ralph CJ, Sauer J, Droege S (1998). Monitoring bird populations by point counts. DIANE Publishing.

[R51] Ramsar Convention on Wetlands, 2018. Global Wetland Outlook: State of the World’s Wetlands and their Services to People. Gland, Switzerland: Ramsar Convention Secretariat. https://www.global-wetland-outlook.ramsar.org/gwo-2018 (accessed 10 May 2025).

[R52] Ramsar Sites Information Service (2023). The List of Wetlands of International Importance. Published 9 May 2023. https://www.ramsar.org/sites/default/files/documents/library/sitelist.pdf

[R53] Rannestad OT, Tsegaye D, Munishi P, Moe S (2015). Bird abundance, diversity and habitat preferences in the riparian zone of a disturbed wetland ecosystem–the Kilombero Valley, Tanzania. Wetlands, 35, 521–532.

[R54] R Core Team (2016). R: A language and environment for statistical computing. R Foundation for Statistical Computing (v4.4.2) (version 4.4.2), Vienna, Austria. http://www.R-project.org/

[R55] Remsen JV Jr. (2020). Greater Thornbird (*Phacellodomus ruber*), version 1.0. In Birds of the World (del Hoyo J., Elliott A., Sargatal J., Christie D. A., and de Juana E., Editors). Cornell Lab of Ornithology, Ithaca, NY, USA. 10.2173/bow.gretho2.01

[R56] Rodgers JA Jr., Smith HT (2020). Little Blue Heron (*Egretta caerulea*), version 1.0. In Birds of the World (Poole A. F., Editor). Cornell Lab of Ornithology, Ithaca, NY, USA. 10.2173/bow.libher.01

[R57] Roper EM (2020). White-collared Swift (*Streptoprocne zonaris*), version 1.0. In Birds of the World (Schulenberg T. S., Editor). Cornell Lab of Ornithology, Ithaca, NY, USA. 10.2173/bow.whcswi.01

[R58] Saccò M, White N, Harrod C, Salazar G (2021). Salt to conserve: a review on the ecology and preservation of hypersaline ecosystems. Biological Reviews, 96(6), 2828–2850. 34747117 10.1111/brv.12780

[R59] Salvador SA, Salvador L, Ferrari C, Vitale S (2016). Checklist of the Birds of Córdoba Province, Argentina. https://www.avesargentinas.org.ar/

[R60] Santos EG, Wiederhecker HC, Lopes LE, Marini MÂ (2023). Equivalence of citizen science and scientific data for modelling species distribution of birds from a tropical savanna. Austral Ecology, 48(8), 2171–2184.

[R61] Schmaljohann H, Eikenaar C, Sapir N (2022). Understanding the ecological and evolutionary function of stopover in migrating birds. Biological Reviews, 97(4), 1231–1252. 35137518 10.1111/brv.12839

[R62] Shealer D, Liechty J, Pierce A, Pyle P, Patten M (2020). Sandwich Tern (*Thalasseus sandvicensis*), version 1.0. In Birds of the World (Billerman S. M., Editor). Cornell Lab of Ornithology, Ithaca, NY, USA. 10.2173/bow.santer1.01

[R63] Skowno AL, Bond WJ (2003). Bird community composition in an actively managed savanna reserve, importance of vegetation structure and vegetation composition. Biodiversity & Conservation, 12, 2279–2294.

[R64] Taft OW, Colwell MA, Isola CR, Safran RJ (2002). Waterbird responses to experimental drawdown: implications for the multispecies management of wetland mosaics. Journal of Applied Ecology, 39(6), 987–1001.

[R65] Toledo M, Quaglia A, Vergara-Tabares D (2018). New sandpiper from an interior sea: confirmation of Curlew Sandpiper (*Calidris ferruginea*) for Argentina. Revista Brasileira de Ornitologia, 26, 214–216.

[R66] Torres RM, Michelutti P (2006). Waterbirds. In: Bucher E.H. (ed.), Bañados del Río Dulce and Mar Chiquita Lake (Córdoba, Argentina). National Academy of Sciences, Córdoba, Argentina, pp 237–249.

[R67] Torres RM, Michelutti P (2007). Bañados del Río Dulce and Mar Chiquita Lake Multiple-Use Reserve. In: Giacomo Di (eds) Important Bird Areas in Argentina: Priority Sites for Biodiversity Conservation. Nature and Conservation Topics No. 5. Aves Argentinas/Argentine Ornithological Association, Buenos Aires, pp 134–137.

[R68] Vivas E (2022). eBird: Online database of bird distribution and abundance [web application]. Cornell Lab of Ornithology, Ithaca, New York. https://ebird.org/checklist/S122598389 (accessed 21 July 2025).

[R69] Vivas E (2023). eBird: Online database of bird distribution and abundance [web application]. Cornell Lab of Ornithology, Ithaca, New York. eBird checklist: https://ebird.org/checklist/S173344782 (accessed 10 May 2025).

[R70] Vivas E (2024). eBird: Online database of bird distribution and abundance [web application]. Cornell Lab of Ornithology, Ithaca, New York. eBird checklist: https://ebird.org/checklist/S158337543 (accessed 10 May 2025).

[R71] Williamson L, Hudson M, O’Connell M, Davidson N, Young R, Amano T, Székely T (2013). Areas of high diversity for the world’s inland-breeding waterbirds. Biodiversity and conservation, 22, 1501–1512.

[R72] Wendeler D (2022). eBird: Online database of bird distribution and abundance [web application]. Cornell Lab of Ornithology, Ithaca, New York. eBird checklist: https://ebird.org/checklist/S124143712 (accessed 21 July 2025).

[R73] Wenny DG, Devault TL, Johnson MD, Kelly D, Sekercioglu CH, Tomback DF, Whelan CJ (2011). The need to quantify ecosystem services provided by birds. The auk, 128(1), 1–14.

[R74] Xu X, Chen M, Yang G, Jiang B, Zhang J (2020). Wetland ecosystem services research: A critical review. Global Ecology and Conservation, 22, e01027.

